# Guanylate Cyclase C Deficiency Causes Severe Inflammation in a Murine Model of Spontaneous Colitis

**DOI:** 10.1371/journal.pone.0079180

**Published:** 2013-11-11

**Authors:** Eleana Harmel-Laws, Elizabeth A. Mann, Mitchell B. Cohen, Kris A. Steinbrecher

**Affiliations:** 1 Department of Pediatrics, University of Cincinnati College of Medicine Cincinnati, Ohio, United States of America; 2 Division of Gastroenterology, Hepatology, and Nutrition, Cincinnati Children’s Hospital Medical Center, Cincinnati, Ohio, United States of America; Duke University Medical Center, United States of America

## Abstract

**Background:**

Guanylate Cyclase C (GC-C; *Gucy2c*) is a transmembrane receptor expressed in intestinal epithelial cells. Activation of GC-C by its secreted ligand guanylin stimulates intestinal fluid secretion. Familial mutations in GC-C cause chronic diarrheal disease or constipation and are associated with intestinal inflammation and infection. Here, we investigated the impact of GC-C activity on mucosal immune responses.

**Methods:**

We utilized intraperitoneal injection of lipopolysaccharide to elicit a systemic cytokine challenge and then measured pro-inflammatory gene expression in colonic mucosa. GC-C^+/+^ and GC-C^−/−^ mice were bred with interleukin (IL)-10 deficient animals and colonic inflammation were assessed. Immune cell influx and cytokine/chemokine expression was measured in the colon of wildtype, IL-10^−/−^, GC-C^+/+^IL-10^−/−^ and GC-C^−/−^IL-10^−/−^ mice. GC-C and guanylin production were examined in the colon of these animals and in a cytokine-treated colon epithelial cell line.

**Results:**

Relative to GC-C^+/+^ animals, intraperitoneal lipopolysaccharide injection into GC-C^−/−^ mice increased proinflammatory gene expression in both whole colon tissue and in partially purified colonocyte isolations. Spontaneous colitis in GC-C^−/−^IL-10^−/−^ animals was significantly more severe relative to GC-C^+/+^IL-10^−/−^ mice. Unlike GC-C^+/+^IL-10^−/−^ controls, colon pathology in GC-C^−/−^IL-10^−/−^ animals was apparent at an early age and was characterized by severely altered mucosal architecture, crypt abscesses, and hyperplastic subepithelial lesions. F4/80 and myeloperoxidase positive cells as well as proinflammatory gene expression were elevated in GC-C^−/−^IL-10^−/−^ mucosa relative to control animals. Guanylin was diminished early in colitis *in vivo* and tumor necrosis factor α suppressed guanylin mRNA and protein in intestinal goblet cell-like HT29-18-N2 cells.

**Conclusions:**

The GC-C signaling pathway blunts colonic mucosal inflammation that is initiated by systemic cytokine burst or loss of mucosal immune cell immunosuppression. These data as well as the apparent intestinal inflammation in human GC-C mutant kindred underscore the importance of GC-C in regulating the response to injury and inflammation within the gut.

## Introduction

Ligand binding to transmembrane guanylate cyclase (GC) receptors initiates cyclic guanosine monophosphate (cGMP) production and activates a variety of cell-type specific signaling cascades [1], [2]. The primary transmembrane GC on epithelial cells of the intestine is guanylate cyclase C (GC-C) which binds peptide ligands present in the lumen of the gut. In the colon, guanylin is the primary GC-C ligand and is produced and secreted predominantly by goblet cells, the epithelial cell type of the intestine that makes and secretes mucus as well as numerous bioactive signaling peptides [3]–[5]. The best characterized effector protein of GC-C-produced cGMP in intestinal epithelial cells is protein kinase G II (PKG II) which regulates the cystic fibrosis transmembrane conductance regulator (CFTR) and Na+ H+ exchanger 3 (NHE3) [6]. Signaling to these membrane channels through GC-C results in ion and water flow into the intestinal lumen and, accordingly, GC-C is thought to be important for luminal hydration of intestinal contents. GC-C and its ligands are relevant to human health in that some forms of infectious *E. coli* target GC-C with superagonist enterotoxins [7]. The resulting deregulated cGMP production elicits uncontrolled fluid secretion and secretory diarrhea.

The GC-C signaling system may also be important to intestinal inflammation in humans. Recently, two separate reports have demonstrated that inheritance of distinct mutations in GC-C lead to altered intestinal fluidity and coincident inflammation. Romi and associates describe a Bedouin kindred with apparent *inactivating* mutations in GC-C that cause small bowel obstruction similar to that seen in cystic fibrosis (CF) [8]. Of note, in addition to non-CF associated meconium ileus, this kindred was originally identified as being prone to gastrointestinal infection during infancy [9]. Conversely, Fiskerstrand et al show that inherited, *activating* mutations in GC-C result in enhanced fluid secretion that often culminates in intestinal inflammation [10]. These seminal reports indicate that GC-C and cGMP production in the epithelial cell layer of the gut has important implications for mucosal responses to injury, infection, and inflammation.

Recent studies by our group and others underscore the complex role of GC-C in intestinal disorders. Mice having the *Gucy2c* gene deleted (GC-C^−/−^) are sensitive to radiation damage as measured by elevated epithelial cell apoptosis [11]. We have also shown that GC-C^−/−^ mice have defective barrier function in the small intestine and that bacterial translocation is enhanced during the stress of intraperitoneal endotoxin challenge [12]. Others have reported similar findings [13]. The impact of barrier dysfunction on intestinal inflammatory disease is context dependent and is heavily influenced by the nature of the barrier defect and the disease model utilized [14]–[16]. We have found that deletion of GC-C provides resistance to colonic injury caused by the ulcerating chemical dextran sodium sulfate (DSS) [16]. Our recent work indicates that infectious colitis caused by the enteric bacteria *Citrobacter rodentium* elicits colonic barrier dysfunction in GC-C^−/−^ mice, allowing systemic spread of the pathogen [17]. In order to further explore the role of GC-C in inflammation of the intestine, we investigated the impact of GC-C deletion on two types of mucosal inflammatory stress, systemic endotoxin challenge and loss of immunosuppressive IL-10.

## Methods

### Mice

Mice with deleted guanylate cyclase C were generated as described and originally provided by Dr. Ralph Giannella of the University of Cincinnati [18]. Heterozygous GC-C^+/−^ mice (*Gucy2c*, guanylate cyclase 2c; GeneID: 14917) mice were repeatedly bred with C57BL/6J (stock #00664) animals obtained from Jackson Laboratories (Bar Harbor, ME, USA). From this process, GC-C^+/+^ wildtype and GC-C^−/−^ knockout mice of >10 generations C57BL/6J genetic background were generated from the same breeding lineage. As in our previously published work, these GC-C^+/+^ and GC-C^−/−^ animals were bred for studies in the same specific pathogen free room within the CCHMC vivarium [12], [16]. IL-10^−/−^ mice (C57BL/6J strain, stock #002251) were obtained from Jackson Laboratories and bred into 10^th^ generation GC-C^−/−^ or GC-C^+/+^ mice. This produced control mouse lines as well as compound transgenic mice lacking IL-10 and GC-C on a pure C57BL/6J background. In these studies, littermate mice were used for analysis and we noted no gene dosage-dependent differences between compound heterozygotes and controls (for example, GC-C^+/−^IL-10^+/−^ versus GC-C^+/+^IL-10^+/+^, nor in GC-C^+/+^IL-10^−/−^ versus GC-C^+/−^IL-10^−/−^). All studies were approved by the Cincinnati Children’s Hospital Medical Center Institutional Animal Care and Use Committee under protocol #1E08069.

### Intraperitoneal Injection of Lipopolysaccharide

Intraperitoneal injections of *E. coli* O55:B5 LPS (Calbiochem, La Jolla, CA, USA) were performed as described in Steinbrecher et al [19]. Briefly, LPS (5 ug/g mouse weight) in saline was injected into the intraperitoneal cavity while sham groups were injected with similar volumes of saline only. Two hours after injection, mice were euthanized and a portion of the colon was frozen for later analysis. The colonic epithelial cell compartment was extracted from the remaining colon tissue using a chelation approach. In a manner similar to that described previously, tissue was incubated in chelation solution (0.5 mM dithiothreitol; 30 mM EDTA in phosphate buffered saline) at 4°C for 30 minutes and then shaken repeatedly to enrich for colonocytes and closely associated lymphocytes [19]. These isolates were immediately processed into RNA using TRIzol reagent (Invitrogen, Carlsbad, CA, USA).

### Realtime RT-PCR

Gene expression analysis using realtime RT-PCR was performed as described previously [16], [19], [20]. RNA was extracted from samples using TRIzol and cDNA was transcribed using Verso cDNA kit (Thermo Fisher Scientific, Pittsburgh, PA, USA). Brilliant II Sybr QPCR (Agilent Technologies, Santa Clara, CA, USA) mix was used to determine gene expression using methods recommended by the manufacturer. The housekeeping gene actin or GAPDH were used to normalize all values. Within each experiment, a single representative wildtype GC-C^+/+^ animal are arbitrarily set at 1 and all other mice are presented relative to this using the ΔΔCt method. All primer sequences have been published or are available upon request [16], [17], [19].

### Histology and Immunofluorescence

Freshly excised intestinal tissue was either fixed in formalin overnight and embedded in paraffin for analysis by H&E, or frozen immediately in OTC for analysis of frozen sections using immunofluorescence. These procedures were performed as reported previously [16], [19], [21]. Myeloperoxidase (RB373A, Thermo Fisher Scientific, Pittsburgh, PA, USA) and F4/80 clone BM8 (14–4801; eBioscience, San Diego, CA, USA) antibodies were incubated at 4°C overnight. Scoring of intestinal histopathology was performed on 5.0 um cross sections of ‘swiss rolled’ colon such that the majority of the tissue was visible to allow for a broad assessment of the disease severity of each animal. Chronic inflammation scoring parameters, as previously reported by us, were based on that of Berg et al and Schultz et al and included measurements of inflammation, epithelial cell hypertrophy, and composition of infiltrating immune cell types [22], [23].

### Cell Culture

The goblet cell like HT29-18-N2 cell line was grown as previously described [24]. Cells were treated with 10 ng/ml TNFα or 100 U/ml IFNγ (Sigma-Aldrich, St. Louis, MO, USA) for 24 hours and then either RNA or protein extracts were processed. Protein concentrations were measured using Bradford assay (Bio-Rad, Hercules, CA, USA) and RNA was processed using TRIzol reagent.

### Western Blot Analysis

As we have previously reported, protein extracts were run on 4–12% denaturing gradient NuPAGE gels (Life Technologies, Carlsbad, CA, USA), blotted onto 0.2 um pore size nitrocellulose and probed overnight at 4°C. Guanylin antibody (Ab#2538) was kindly provided by Dr. Michael Goy of the University of North Carolina, Chapel Hill. Antibody specific to β-tubulin (SC-9104) was obtained from Santa Cruz Biotechnology (Santa Cruz, CA, USA).

### Statistical Analysis

Data are presented as mean with SEM and was analyzed using the Mann-Whitney or 2-tailed Student *t* tests. Calculations were performed using Prism Version 5.03 (GraphPad Software, Inc., San Diego, CA) and statistical significance was set at p≤0.05.

## Results

### GC-C Deficiency Enhances Colonic Cytokine Production during LPS Challenge

Diminished production of epithelial cGMP due to loss of GC-C activity results in barrier dysfunction and tight junction disassembly during experimental systemic LPS exposure. In addition, there is an increase in circulating cytokines such as IFNγ in GC-C^−/−^ mice relative to control animals [12]. Accordingly, we speculated that the GC-C^−/−^ intestine may be more sensitive to some forms of pro-inflammatory stimuli. To test this, we challenged control and GC-C^−/−^ mice with intraperitoneal injection of LPS. This results in a rapid elevation in circulating pro-inflammatory cytokines such as TNFα and IFNγ. We and others have used this approach as a way of gauging the response of the intestinal mucosa to immune cell activation and cytokine-induced stress as opposed to that caused by direct chemical or radiation injury [19], [25]. We used realtime RT-PCR to measure gene expression in whole colonic tissue from control and GC-C^−/−^ mice two hours after intraperitoneal LPS injection. Analysis of whole colonic tissue revealed that GC-C^−/−^ mice were highly responsive and strongly expressed cytokines, chemokines, and immunoregulatory genes, including IFNγ, IL-17, IL-22, CXC motif-like (CXCL)5/9/10, and indoleamine 2,3-dioxygenase 1 to a greater degree than wildtype animals **(** **Table 1** **)**. We then performed similar experiments in which gene expression was analyzed in the colonic epithelial cell compartment. We noted that pro-inflammatory gene expression in partially purified GC-C^−/−^ colonocytes was much higher than GC-C^+/+^ **(** **Table 2** **)**. Importantly, genes critical for epithelial-regulated mucosal immune responses, such as CXCL5/9/10 and thymic stromal lymphopoietin (TSLP), were significantly elevated. Antimicrobial genes expressed by epithelial cells and closely associated γδ intraepithelial lymphocytes were elevated as well and included regenerating islet-derived 3β (RegIIIβ) and RegIIIγ. While we have primarily focused on gene expression responses in the colon, we also found that small bowel epithelial cell gene expression was affected by LPS challenge (CXCL5 WT 52.7±2.1 vs. GC-C^−/−^279.3±94.4, p = 0.02; TSLP WT 21.13±6.7 vs. GC-C^−/−^120.9±64.2, p = 0.03). These studies clearly indicated that systemic circulating cytokines elicit a robust and heightened gene expression response in the colon in the absence of GC-C. Furthermore, this implied that loss of GC-C may exacerbate inflammation in intestinal disease models that are initiated by deregulated cytokine expression by immune cells.

**Table 1:** LPS-induced gene expression in colon of GC-C wildtype and GC-C null mice.

Colon	GC-C WT	GC-C null	p (t test)
**CXCL1**	85±1.4	76±12	0.5
**CXCL5**	17±1.4	33±6.2	0.04
**CXCL9**	2.9±0.11	8.0±0.7	0.02
**CXCL10**	34±2.6	62±11	0.05
**CCL2/MCP-1**	173±23	196±12	0.4
**IFNγ**	4.4±0.57	11±2.7	0.04
**TNFα**	23±5.4	25±4.1	0.8
**IL-1β**	12±3.1	7.3±1.4	0.2
**IL-6**	73±8.4	85±11	0.4
**IL-12p40**	1.5±0.38	2.1±0.4	0.2
**IL-17A**	8.8±1.0	23±2.2	0.02
**IL-17F**	7.1±0.5	18±3.0	0.07
**IL-18**	3.0±0.5	2.5±0.3	0.5
**IL-22**	70±8.7	139±18	0.01
**IDO**	1.5±0.6	23±4.8	0.04

120′ post-LPS; all values are mean±SEM and are relative to WT untreated;

n = 4–6/group.

**Table 2:** LPS-induced gene expression in colonocytes of GC-C wildtype and GC-C null mice.

Colon IEC	GC-C WT	GC-C null	p (t test)
**CXCL1**	124±48	116±9.0	0.8
**CXCL3**	4.1±2.3	8.7±3.5	0.2
**CXCL5**	19±1.7	67±13	0.01
**CXCL9**	24±6.2	170±15	0.01
**CXCL10**	190±19	755±81	0.0005
**CCL2/MCP1**	107±20	159±12	0.1
**CCL3/MIP-2**	3.7±0.7	6.2±2.0	0.4
**TSLP**	19±7.9	245±75	0.05
**IL-22R**	9.6±3.4	11±2.5	0.7
**RegIIIα**	5.7±2.2	13±6.7	0.3
**RegIIIβ**	0.5±0.04	16±1.7	0.0007
**RegIIIγ**	6.3±4.2	19±4.2	0.07
**S100A8**	162±81	118±4.9	0.6
**S100A9**	137±74	82±7.1	0.5
**Bcl-xL**	1.1±0.11	0.84±0.27	0.3
**cIAP2**	4.1±1.4	2.5±0.1	0.4
**XIAP**	3.9±2.2	4.7±2.9	0.8

120′ post-LPS; all values are mean±SEM and are relative to WT untreated;

n = 4/group.

### Accelerated Onset of Severe Colonic Inflammation in Mice Lacking both GC-C and IL-10

We have shown that chemical initiators of colitis that act on the epithelial cell layer, such as DSS, cause less disease in mice lacking GC-C. This is due, at least in part, to altered goblet cell gene expression [16]. The DSS experimental colitis model is highly dependent on goblet cell numbers within the colon as well as specific goblet cell gene products which differentially regulate disease severity or resistance [14], [15], [26], [27]. We have previously hypothesized that GC-C^−/−^ mice would be susceptible to some forms of intestinal inflammation and this is supported by our current LPS challenge studies ([16] and Tables I and II). Therefore, we next chose to examine the response of GC-C^−/−^ animals to spontaneous intestinal inflammation using a system that more closely models human disease. The IL-10^−/−^ mouse model of colitis is initiated by loss of T cell immunosuppression that is exacerbated by commensal microflora and intestinal barrier dysfunction [23], [28], [29]. We hypothesized that the hyper-responsive state of the GC-C^−/−^ colonic mucosa to immune cell activation would result in enhanced disease in the context of T cell-dependent colitis such as that which occurs during IL-10^−/−^ deficiency.

**Figure 1:**
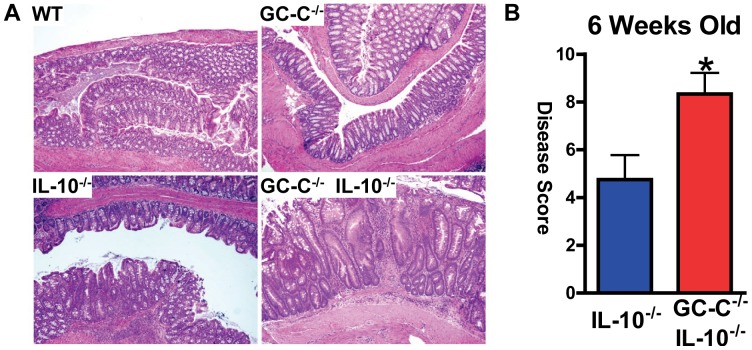
Deletion of GC-C accelerates the development of severe colitis in IL-10 deficient 6 week old mice. (A) Histological analysis of H&E stained colon clearly demonstrates that mice lacking both GC-C and IL-10 have severe colitis characterized by inflammatory cell infiltrate and epithelial hyperplasia. (B) Scoring of disease parameters indicates that GC-C^−/−^IL-10^−/−^ mice have more severe colonic inflammation. n = 3–4 mice per group; *p = 0.05.

**Figure 2:**
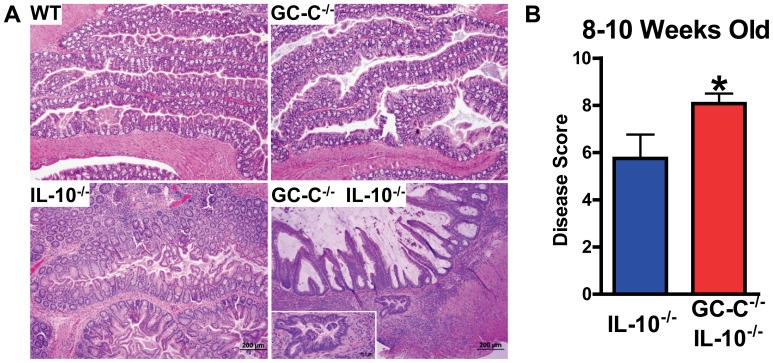
GC-C^−/−^IL-10^−/−^ mice have severe colitis and pre-cancerous lesions at 8–10 weeks of age. (A) While mice lacking IL-10 alone had moderate inflammatory disease at 8–10 weeks of age, GCC^−/−^IL-10^−/−^ were severely affected and had clear indication of progression toward adenocarcinoma (inset). (B) Disease scores confirm that there is significantly more pathology in GC-C/IL-10 double knockout mice. n = 8–14 mice per group; *p<0.03.

We bred mice deficient in GC-C with IL-10^−/−^ animals and developed GC-C^−/−^IL-10^−/−^ and control GC-C^+/+^IL-10^−/−^ mice that were >10 generations into the C57BL/6J genetic background. As expected, we found no indication of inflammation in wildtype or GC-C^−/−^ colon. IL-10^−/−^ mice had colitis that was mild by 6 weeks of age in CCHMC specific pathogen free vivarium. However, we found that loss of GC-C results in the accelerated appearance of colitis in IL-10^−/−^ animals. Compound knockout GC-C^−/−^IL-10^−/−^ mice presented with obvious signs of gastrointestinal disease, including diarrhea and rectal prolapse, by 6 weeks of age. Histological analysis indicated that GC-C^−/−^IL-10^−/−^ animals had severe epithelial hyperplasia and apoptosis, frequent crypt abscesses, and significant mixed inflammatory infiltrate **(** **Figure 1A** **)**. Crypt-surface architecture was massively disrupted in these mice and transmural inflammation was common. Histological disease scoring confirmed that mice lacking both GC-C and IL-10 develop more severe disease by six weeks of age **(** **Figure 1B** **)**. We also analyzed 8–10 week old mice and found, as expected, that GC-C^−/−^IL-10^−/−^ mice continued to have more significant disease as compared to IL-10^−/−^ mice **(** **Figure 2A** **)**. Notably, in addition to profound transmural inflammation and disruption of normal epithelial crypt-surface placement, many of the more severely affected GC-C^−/−^IL-10^−/−^ mice had developed clear indications of inflammation-associated epithelial transformation and progression toward adenocarcinoma with frequent gland penetration into the intestinal musculature **(** **Figure 2A** **, inset)**. IL-10^−/−^ mice of this age did not display similar precancerous lesions. Disease scoring clearly indicated that GC-C^−/−^ mice that lack IL-10 have significantly more intestinal inflammation as compared to animals lacking only IL-10 **(** **Figure 2B** **)**.

**Figure 3:**
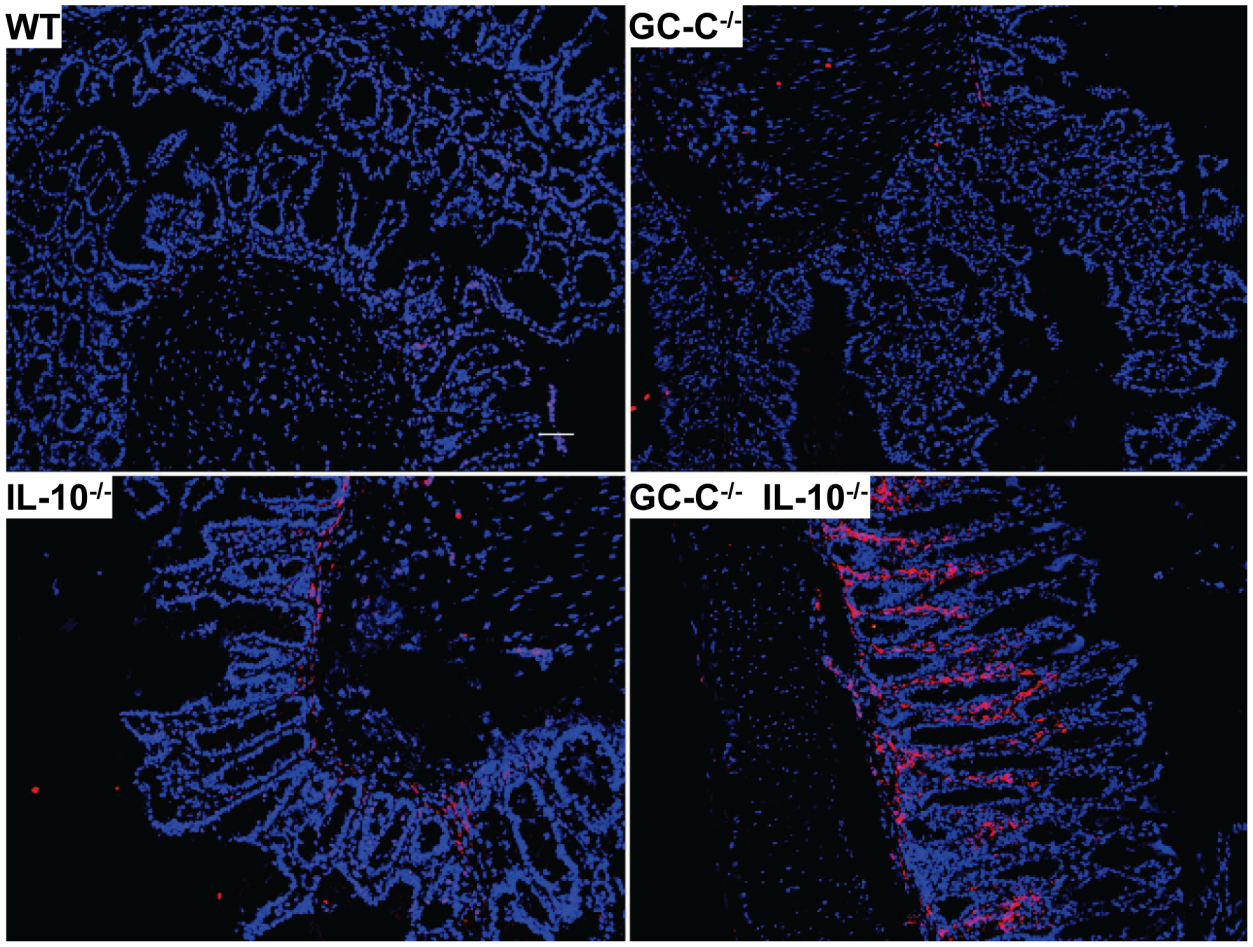
Infiltration of F4/80+ cells into the colon is greatly increased in the absence of GC-C and IL-10. Representative images show that while there are increased F4/80+ cells in IL-10^−/−^ colon, most of which are likely macrophages, there is far greater staining in the severely diseased colon of GC-C^−/−^IL-10^−/−^ mice. F4/80 is depicted in red while nuclei are stained blue using DAPI. Magnification = 200X.

Colitis in IL-10^−/−^ mice is characterized by substantial inflammatory cell movement into the intestinal mucosa that consists of a variety of cell types including macrophages and neutrophils. We next investigated macrophage and neutrophil infiltration in 6 week old IL-10^−/−^ and GC-C^−/−^IL-10^−/−^ mice using F4/80 and myeloperoxidase (MPO) as markers, respectively. No differences were noted in baseline macrophage or neutrophil numbers in wildtype controls relative to GC-C^−/−^ mice. Consistent with the histological findings in Figure 1A, we found substantially more staining of both F4/80 and MPO in GC-C^−/−^IL-10^−/−^ colon as compared to IL-10^−/−^ **(** **Figures 3** **and** **4** **)**. Collectively, these data clearly show that loss of GC-C leads to early inflammation in an IL-10^−/−^ setting that is characterized by increased inflammatory cell infiltration, distortion of mucosal architecture, and pre-cancerous epithelial cell morphology.

**Figure 4:**
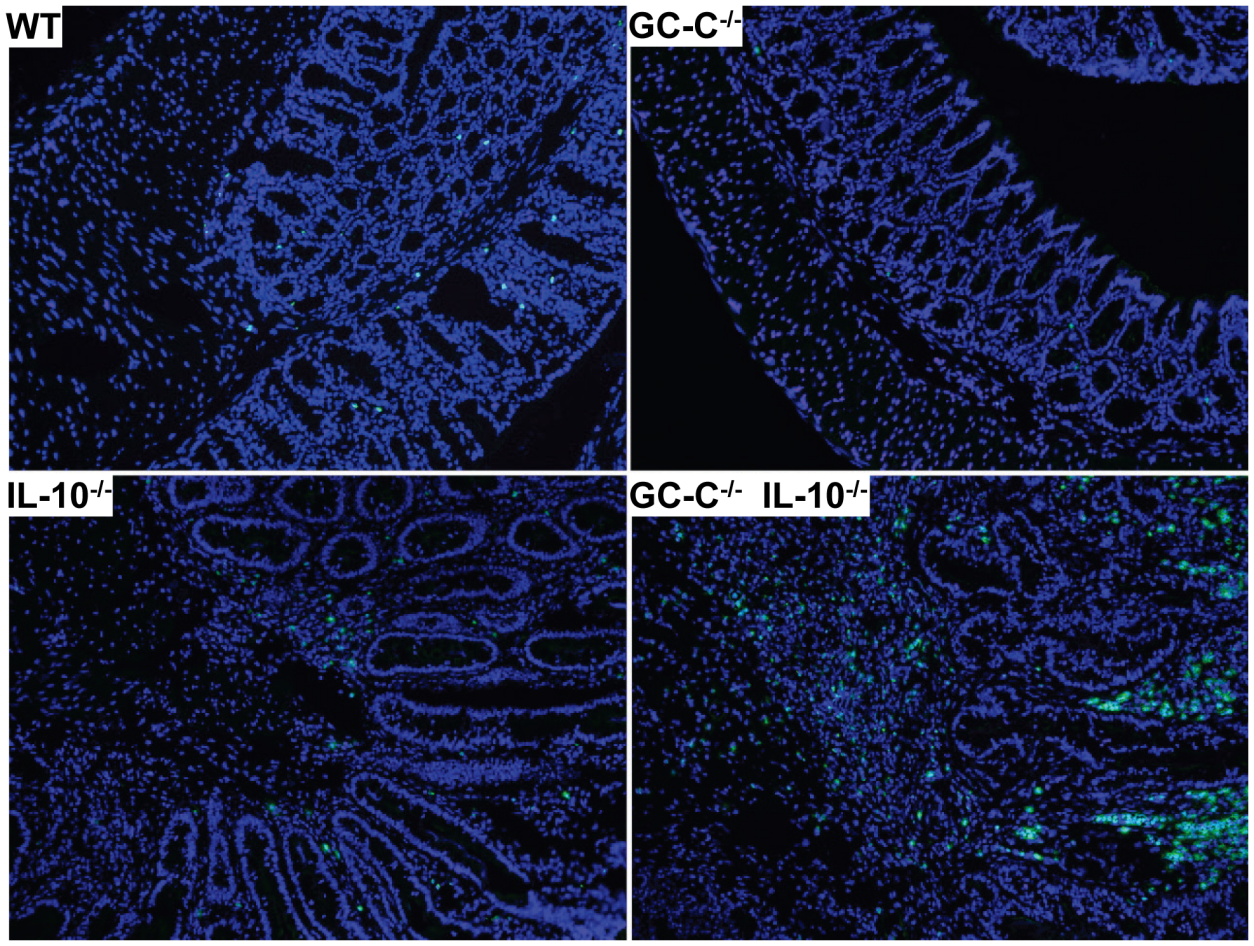
Myeloperoxidase positive cells are present in much greater numbers in GC-C^−/−^IL-10^−/−^ colonic tissue as compared to IL-10^−/−^. Representative images showing greater numbers of MPO stained cells, most of which are neutrophils, in colon lacking GC-C and IL-10. Myeloperoxidase is depicted in green while nuclei are stained blue using DAPI. Magnification = 200X.

### Loss of GC-C Increases Colonic Cytokine Production in IL-10^−/−^ Mice

**Figure 5:**
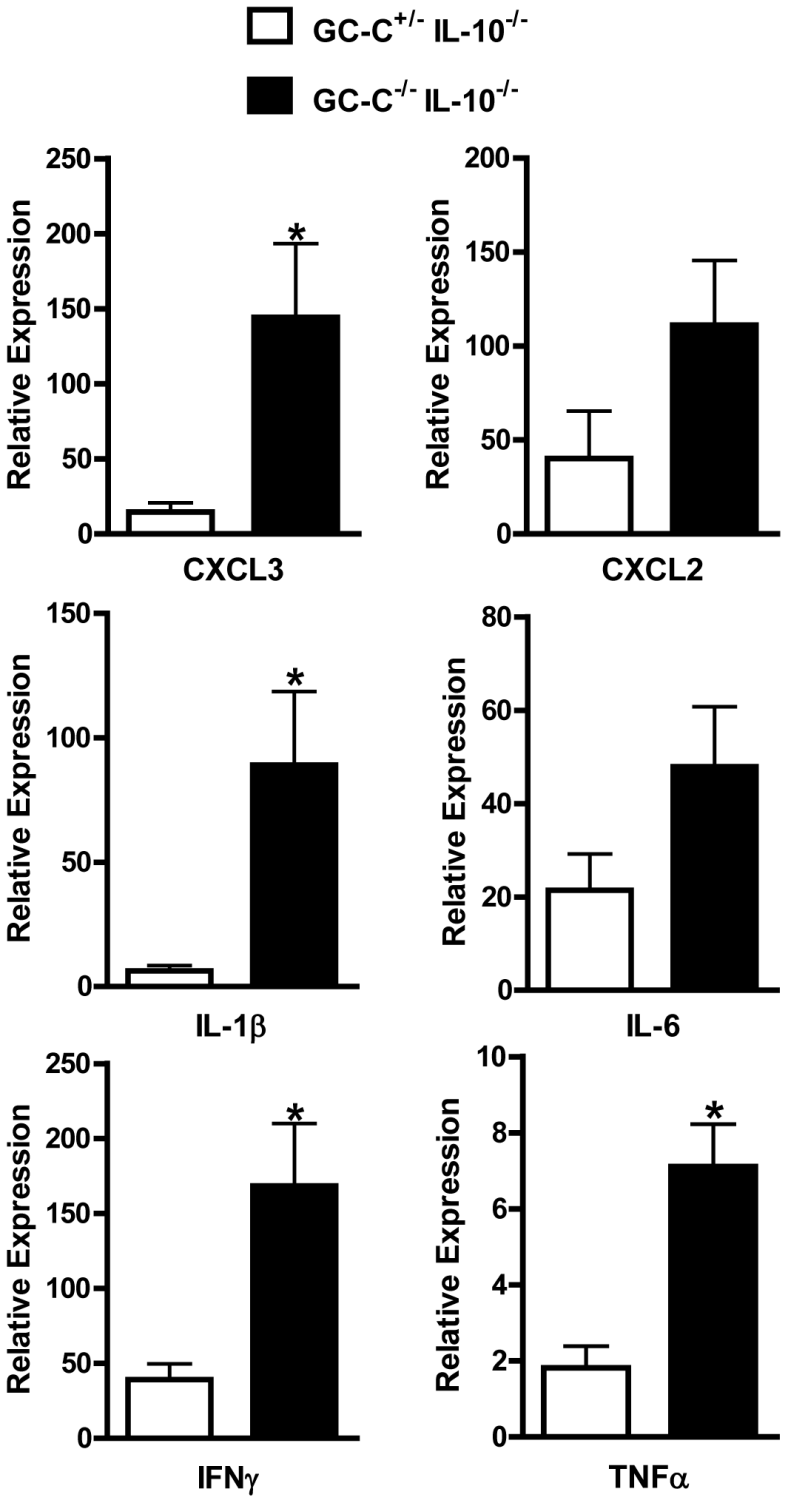
Analysis of pro-inflammatory gene expression using realtime RT-PCR demonstrates enhanced cytokine and chemokine production in GC-C^−/−^IL-10^−/−^ tissue. Gene expression in colon of IL-10^−/−^ and GC-C^−/−^IL-10^−/−^ mice are shown relative to wildtype colon which is arbitrarily set at 1. n = 4–12 mice per group; *p<0.05.

We anticipated that elevated levels of cytokines would be present during colitis in GC-C^−/−^IL-10^−/−^ mice. We used realtime RT-PCR to analyze colonic gene expression in mice greater than 8 weeks of age and found that GC-C^−/−^IL-10^−/−^ mice had substantial increases in multiple chemokines including CXCL2 and CXCL3 as well as pro-inflammatory cytokines such as TNFα, IFNγ, IL-1β, IL-6 **(** **Figure 5** **)**. We measured no differences in expression of these genes in GC-C^−/−^ tissue relative to wildtype controls (data not shown). These gene expression data along with the above histological findings reveal that GC-C signaling is an important modifier of T-cell driven chronic colitis in mice.

### Guanylin, the Primary GC-C-activating Ligand in the Colon, is Decreased in Colitis

**Figure 6:**
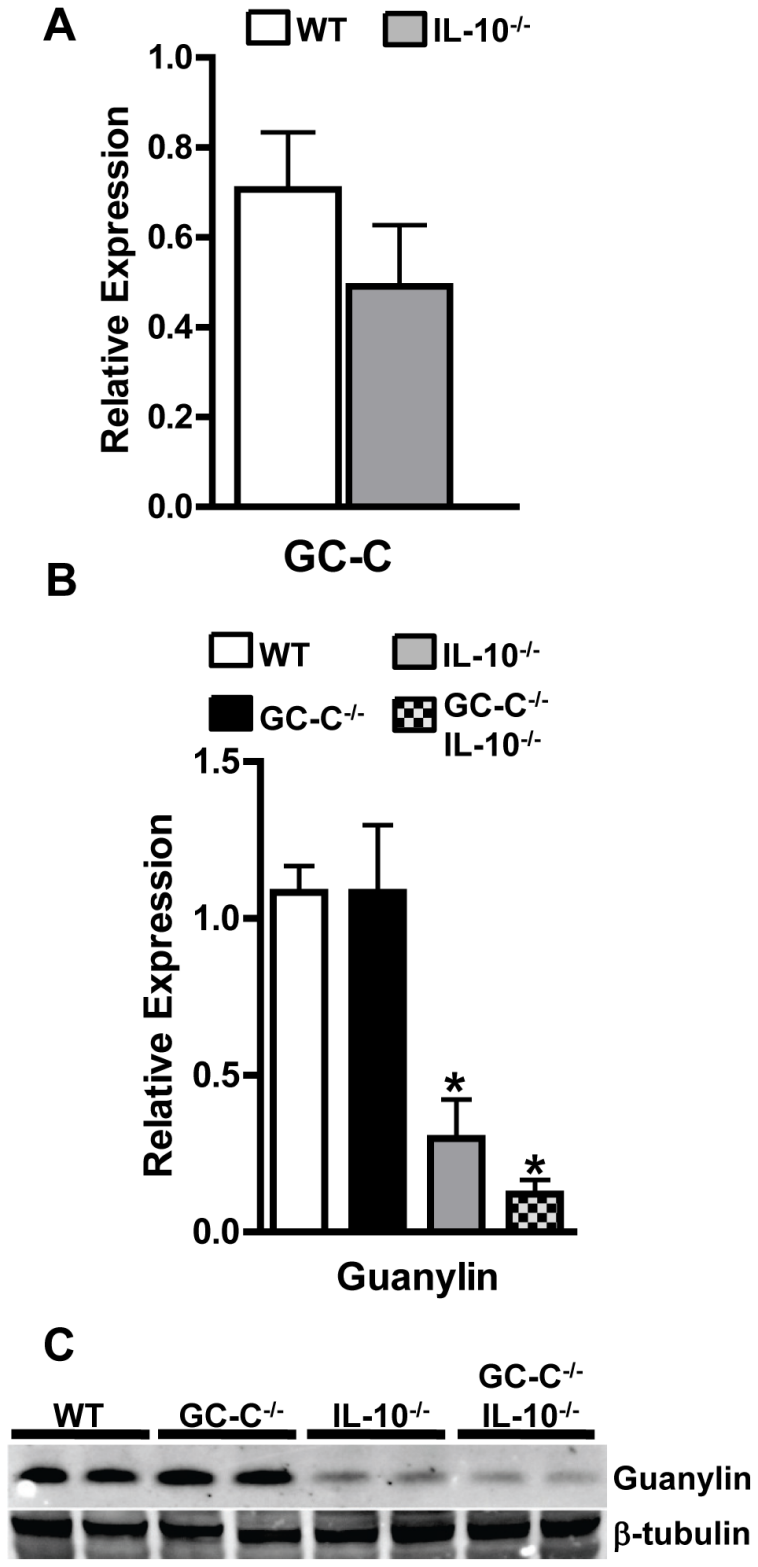
Guanylin production is nearly absent during colitis associated with IL-10 deficiency. (A) GC-C mRNA is not affected by intestinal inflammation, as measured by realtime RT-PCR. n = 5–6 per group (B) Guanylin gene expression is highly reduced in GC-C^−/−^IL-10^−/−^ colon. n = 5 per group; *p<0.005 (C) Western blotting indicates that guanylin protein expression is suppressed by colonic inflammation in both IL-10^−/−^ and GC-C^−/−^IL-10^−/−^ mice. On this representative blot, each lane represents a sample from an individual mouse. β-tubulin is shown to demonstrate equal loading within each lane.

Recent studies indicate that familial polymorphisms in the GC-C gene may be important for susceptibility to intestinal disorders including intestinal inflammation [8], [10]. Our data in GC-C^−/−^IL-10^−/−^ mice indicates that loss of GC-C signaling influences the timing and severity of immune cell-mediated colitis. We next investigated whether the converse was also true, that is, if intestinal inflammation influenced the expression of GC-C or its primary colonic ligand, guanylin. Relative to wildtype mice, realtime RT-PCR indicated that GC-C expression was not changed in adult IL-10^−/−^ mice with active colitis **(** **Figure 6A** **)**. Guanylin is produced in goblet cells of the intestine and goblet cell depletion often accompanies the latter, more severe stages of intestinal inflammation. IL-10^−/−^ mice which had moderate colitis and only slight goblet cell loss had significantly diminished guanylin mRNA levels **(** **Figure 6B** **)**. As expected, guanylin expression was greatly reduced in severely affected GC-C^−/−^IL-10^−/−^ animals that had significant goblet cell depletion. Diminished guanylin production was even more evident in the analysis of colonic protein extracts using western blotting which demonstrated substantial loss of guanylin protein in both IL-10^−/−^ as well as GC-C^−/−^IL-10^−/−^ colon **(** **Figure 6C** **)**. We speculate that the progressive loss of guanylin during inflammation leads to diminishing activation of GC-C and that this may be linked to disease severity in spontaneous models of murine colitis.

### Guanylin Expression is Suppressed by TNFα in vitro

**Figure 7:**
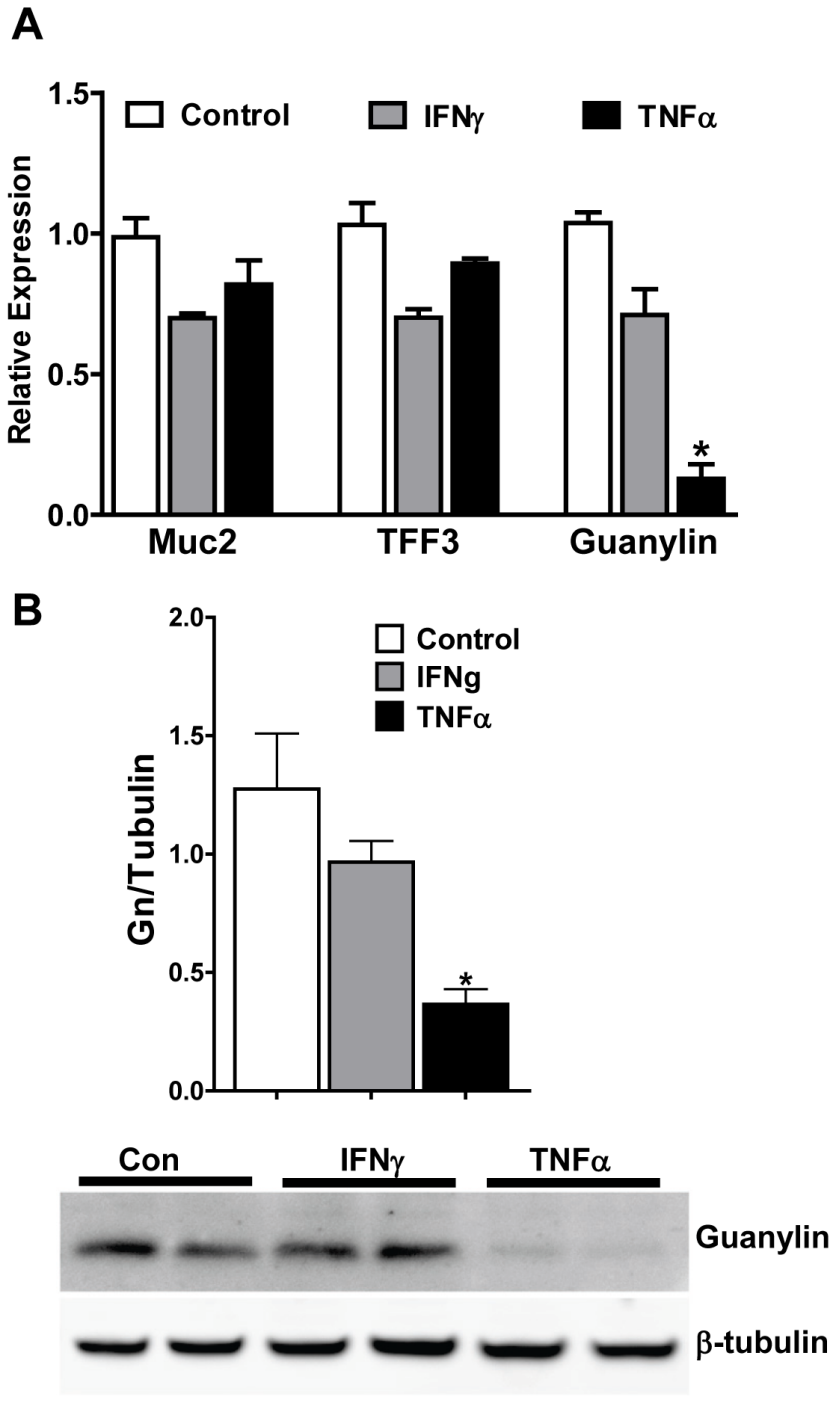
TNFα suppresses guanylin expression. Goblet cell-like HT29-18-N2 cells were treated with 10 ng/ml TNFα or 100 U/ml IFNγ for 24 hours and guanylin mRNA and protein were measured. (A) Realtime RT-PCR analysis indicated that, unlike other goblet cell genes such as Muc2 and TFF3, guanylin expression was substantially depressed by TNFα. n = 4 per group; *p<0.05 (B) A representative western blot of guanylin protein, as well as quantitation of multiple blots from independent experiments, shows that guanylin protein is greatly decreased following TNFα exposure but is not affected by IFNγ. n = 8 individual samples per group, *p<0.05.

We noted that guanylin production was reduced in IL-10^−/−^ mice that had histologically mild to moderate colitis and very little goblet cell ablation. Cytokine levels, however, were high at these analysis timepoints leading us to hypothesize that guanylin expression may be negatively affected by exposure to cytokines. We therefore utilized a reductionist system in order to investigate the specificity of diminished guanylin mRNA in response to cytokine challenge. HT-29-18-N2 cells are a human cell line that has been used by us and others as a goblet cell-like culture model that expresses guanylin mRNA and protein [24]. We exposed these cells to two candidate cytokines, IFNγ and TNFα, and determined guanylin expression using realtime RT-PCR after 24 hours. We also analyzed expression of Mucin 2 (Muc2) and trefoil factor 3 (TFF3), two genes specifically expressed in goblet cells [30], [31]. We noted that Muc2 and TFF3 levels were mildly decreased by IFNγ or TNFα treatment but that this difference did not meet statistical significance **(** **Figure 7A** **)**. Guanylin mRNA, although only mildly affected by exposure to IFNγ, was strikingly suppressed by TNFα treatment. Using western blot analysis, we found that guanylin protein was also greatly diminished following 24 hours of TNFα treatment. Quantitative densitometry of western blots from multiple experiments demonstrated a substantial reduction in guanylin protein production in TNFα-treated cells **(** **Figure 7B** **)**. Based on these data, the presence of TNFα during the early stages of colitis may have the important effect of suppressing guanylin production and reducing GC-C signaling activity.

## Discussion

Several human kindreds with mutations in the *Gucy2c* gene have recently been reported. Romi and colleagues identified two separate families with apparent non-cystic fibrosis meconium ileus [8], [9]. An inactivating mutation in the coding region of the GC-C gene implicated reduced cGMP-regulated ion and water flow into the intestinal lumen as the basis for thickened meconium and obstruction. Conversely, Fiskerstrand et al identified a family in which the typical, initial presentation was chronic diarrhea accompanied by electrolyte imbalances (metabolic acidosis, hyponatremia) during the neonatal period [10]. An activating mutation in GC-C was identified that generates several fold more cGMP upon activation versus wildtype. Intestinal disorders in these affected individuals likely stem from GC-C/cGMP-enhanced ion movement and include inflammatory bowel disease (specifically Crohn’s disease), intestinal obstruction associated with volvulus, and infectious gastroenteritis. Collectively, these studies establish GC-C-dependent electrolyte and water secretion into the bowel as essential to gastrointestinal health and underscore the physiological importance of increased GC-C expression and receptor numbers during the neonatal period [32], [33].

Similar to that shown in human patients with GC-C mutation, we show here that deletion of GC-C in mice results in rapidly developing, severe colitis in a spontaneous intestinal inflammation model (IL-10^−/−^ mice) that closely resembles human inflammatory bowel disease (IBD). As is likely the case in IBD, the spontaneous intestinal pathology in IL-10^−/−^ mice is driven by loss of immunosuppression of T-cells that is exacerbated by epithelial barrier dysfunction via entry of luminal antigens into the subepithelial compartment. Small bowel barrier defects in IL-10^−/−^ mice begin at the weaning period and prior to histopathology [28], [29]. Pharmacological interventions that increase barrier function in these mice delay the on-set and reduce the severity of colitis [28], [29]. We and others have demonstrated that loss of GC-C signaling results in intestinal barrier dysfunction [12], [13], [17]. Based on available data, it seems likely that the early appearance and increased severity of inflammation in GC-C^−/−^IL-10^−/−^ mice is not due to an active, direct role for GC-C in suppressing immune cell activation but is caused by loss of GC-C-dependent epithelial barrier function. However, we cannot rule out other possible mechanisms at this time. Recent work suggests that GC-C may regulate food intake, obesity, and activity level and these may impact the severity of inflammation in the GC-C^−/−^IL-10^−/−^ intestine [34], [35]. GC-C-dependent signaling pathways that regulate epithelial cell proliferation or differentiation may also be relevant. We noted numerous pre-cancerous lesions in GC-C^−/−^IL-10^−/−^ mice but further work is required to determine if enhanced epithelial hypertrophy is due to an intrinsic property of GC-C^−/−^ cells, or is simply a response to the prolonged, severe inflammation that develops in GC-C^−/−^IL-10^−/−^ mice [23]. Nonetheless, the reported intestinal phenotypes of GC-C^−/−^ mice, including barrier dysfunction, may be a secondary response to subtle but physiologically relevant deregulation of epithelial ion/water flow.

While the present study indicates that GC-C blunts the severity of mucosal inflammation in the IL-10^−/−^ murine IBD model, we have previously shown that GC-C^−/−^ mice are resistant to DSS-induced epithelial ulceration and colonic injury [16]. Genetic background variability is not an issue in the current or previous reports because they were performed with GC-C^+/+^ and GC-C^−/−^ mice bred through >10 backcrosses into C57Bl/6J and housed in the same room in our vivarium [12], [16], [17]. Importantly, the primary, initiating mechanism of DSS-mediated inflammation is widespread epithelial ulceration and epithelial barrier dysfunction does not always correlate with increased injury in this model. A relevant example is mice lacking the goblet cell protein resistin-like molecule β (RELMβ) which, despite having enhanced intestinal permeability, are resistant to DSS-induced inflammation but sensitive to T cell-dependent colitis [14], [27] We have shown that RELMβ expression is very low in the colon of unchallenged GC-C^−/−^ animals and that colonic instillation of RELMβ normalizes the response of GC-C^−/−^ mice to DSS injury [16]. Recently, Lin et al reported increased sensitivity to DSS colonic injury in GC-C deficient [13], [36]. Inclusion of both sexes in their DSS studies make interpretation of their work difficult, as the sex-dependent response to DSS is well recognized and documented [19], [37]–[39]. Notably, mice lacking PKG II, an important effector protein in the GC-C signaling pathway, have a similar response to DSS as control animals [40]. While additional work is necessary to clarify the role for GC-C in a chemical-induced ulcerating injury approach, we have used a disease model that closely resembles human IBD to demonstrate an important role for this receptor in suppressing spontaneous colitis.

We have also shown that GC-C regulates mucosal cytokine expression during systemic endotoxin challenge. Similar to our findings in the case in GC-C^−/−^IL-10^−/−^ mice, animals lacking GC-C cannot properly respond to strong activation of the immune system following exposure to LPS. We have previously shown intestinal barrier dysfunction in GC-C^−/−^ mice at baseline and following intraperitoneal LPS [12]. Further, we have recently demonstrated significant colonic barrier dysfunction in GC-C^−/−^ mice following enteric bacterial infection [17]. Whether it be endotoxin challenge, bacterial infection, or colitis due to the absence of IL-10, compromised barrier function and entry of luminal material into the subepithelial compartment may activate resident submucosal cell types and result in exacerbated mucosal cytokine production. In addition, deranged signal transduction intrinsic to GC-C^−/−^ cells may enhance pro-inflammatory gene expression in epithelia. Reports indicate that AKT and cSrc are improperly regulated in GC-C-deficient intestinal cells and this may influence cytokine-induced gene expression [41], [42]. Deregulation of ion transporters downstream of GC-C may also regulate proinflammatory pathways, as exemplified by elevated Nuclear factor-kB in cells with compromised CFTR activity [43]–[45]. Further studies will be necessary to define the precise mechanism of enhanced gene expression in GC-C^−/−^ mucosa in response to cytokine challenge.

Here, we also show that TNFα is a strong suppressor of guanylin expression. *In vitro* studies indicate that guanylin production is specifically and profoundly diminished by TNFα and this is consistent with the loss of guanylin expression IL-10^−/−^ colitis. This may have important implications for the development and progression of mucosal inflammation. TNFα is highly expressed at early stages of colitis and may effectively reduce GC-C activation in the colon as the disease progresses. Loss of GC-C signaling could in turn further exacerbate inflammation through barrier dysfunction or enhanced cytokine production. In the present study, genetic deletion of GC-C may synergize with loss of IL-10 at an early age to accelerate the appearance and progression of colitis.

## Conclusion

Recent work clearly establishes GC-C signaling during the neonatal period as an essential component of gastrointestinal electrolyte and fluid homeostasis. In addition to disorders associated with deregulated luminal hydration, a consequence of *Gucy2c* mutation is susceptibility to IBDs such as Crohn’s disease. We have demonstrated using a murine model of IBD that GC-C is an important suppressor of spontaneous, T cell-dependent intestinal inflammation. A better understanding of GC-C/cGMP-regulated signaling networks will be necessary to define the mechanism by which ion movement in the intestine impacts barrier function, as well as other aspects of epithelial monolayer function, to suppress intestinal inflammation.
